# Representation of Time-Relevant Common Data Elements in the Cancer Data Standards Repository: Statistical Evaluation of an Ontological Approach

**DOI:** 10.2196/medinform.8175

**Published:** 2018-02-22

**Authors:** Henry W Chen, Jingcheng Du, Hsing-Yi Song, Xiangyu Liu, Guoqian Jiang, Cui Tao

**Affiliations:** ^1^ The University of Texas at Austin Austin, TX United States; ^2^ School of Biomedical Informatics The University of Texas Health Science Center at Houston Houston, TX United States; ^3^ Mayo Clinic College of Medicine Rochester, MN United States

**Keywords:** common data elements, database management systems, database, time, biomedical ontology

## Abstract

**Background:**

Today, there is an increasing need to centralize and standardize electronic health data within clinical research as the volume of data continues to balloon. Domain-specific common data elements (CDEs) are emerging as a standard approach to clinical research data capturing and reporting. Recent efforts to standardize clinical study CDEs have been of great benefit in facilitating data integration and data sharing. The importance of the temporal dimension of clinical research studies has been well recognized; however, very few studies have focused on the formal representation of temporal constraints and temporal relationships within clinical research data in the biomedical research community. In particular, temporal information can be extremely powerful to enable high-quality cancer research.

**Objective:**

The objective of the study was to develop and evaluate an ontological approach to represent the temporal aspects of cancer study CDEs.

**Methods:**

We used CDEs recorded in the National Cancer Institute (NCI) Cancer Data Standards Repository (caDSR) and created a CDE parser to extract time-relevant CDEs from the caDSR. Using the Web Ontology Language (OWL)–based Time Event Ontology (TEO), we manually derived representative patterns to semantically model the temporal components of the CDEs using an observing set of randomly selected time-related CDEs (n=600) to create a set of TEO ontological representation patterns. In evaluating TEO’s ability to represent the temporal components of the CDEs, this set of representation patterns was tested against two test sets of randomly selected time-related CDEs (n=425).

**Results:**

It was found that 94.2% (801/850) of the CDEs in the test sets could be represented by the TEO representation patterns.

**Conclusions:**

In conclusion, TEO is a good ontological model for representing the temporal components of the CDEs recorded in caDSR. Our representative model can harness the Semantic Web reasoning and inferencing functionalities and present a means for temporal CDEs to be machine-readable, streamlining meaningful searches.

## Introduction

### Background

With a burgeoning volume of heterogeneous data within the field of health care, health informatics research has focused on finding efficient ways to handle the large influx of new data [1]. One approach is to adopt models to standardize and normalize health care data for efficient data integration and sharing. However, a vast proportion of upwards to 80% of electronic clinical data remains unstructured [2]. Recent efforts on standard terminologies and information models such as Systematized Nomenclature of Medicine—Clinical Terms, *Logical Observation Identifiers Names and Codes*, and OpenEHR archetypes have demonstrated the move toward structuralized electronic health data [3-5].

Semantic interoperability has especially been a key goal of health care systems. Specifically, improvements to the quality and cost of health care are the primary reasons for achieving semantic interoperability within the health care system [6]. Approximately 16% of all reported errors in clinical care are attributed to missing information in patients’ electronic health record (EHR) [7]. Additionally, there exists a high level of waste within the health care system [8]. Although a high proportion of the waste comes from the practice of defensive medicine, a significant fraction, constituting $40 million of waste at a single hospital system annually, is the fruit of excessive and unnecessary testing that is the result of the lack of semantic interoperability [9].

Achievement of semantic interoperability has been pursued via representation in the Semantic Web primarily because of its ability to represent the varied features of temporal data. Numerous ontologies have been developed in the recent past, such as CHRONOS, PSI-time ontology, and Resource State/Condition Description Framework ontology [10-12]. Upon reviewing these ontologies, it has been found that overall, these ontologies are lacking in certain key features such as time phase and modality [13]. Additionally, these ontologies were primarily created for general temporal representation and do not specifically address the minutiae of clinical applications. A recently developed ontology, the Time Event Ontology (TEO), addresses the aforementioned shortcomings [14]. TEO, being geared toward temporal annotations in clinical contexts, is utilized and examined in this paper.

There is also a specific need to model temporal relationships within EHRs. In clinical research, time plays an important role in many studies. Temporal reasoning and temporal data management have been identified as two directions of research that are important and relevant to designing architectures for representing the temporal dimension [9]. Temporal reasoning involves the creation of inferred temporal relations between various events. Temporal data maintenance handles the repository of temporal data and the querying of the repository. By modeling temporal relationships with these approaches, study of the time dimension in clinical data becomes possible. For example, careful study of the temporal dimension allows for the elucidation of disease progressions and cause-effect relationships within a clinical setting based on temporal precedent [13].

Current state-of-the-art work in clinical information modeling and extraction includes the HL7 V3 and OpenEHR. Both conform to the ISO 8601 standard as the basis of their syntax [15]. The HL7 V3 represents time based on the following five defined classes: point in time, interval, duration, periodic time, and periodic time as sets [16]. The last class allows HL7 V3 to represent cumulative periodic times. OpenEHR utilizes date, time, date-time, and duration data types [17,18]. OpenEHR allows fields to be missing, allowing for modality to be modeled within the temporal data. These two standardized clinical models can robustly represent temporal data, with each model having its strengths and weaknesses. Unfortunately, these models are only applicable to structured data, leaving out the vast majority of data that is unstructured.

Common data elements (CDEs) have been implemented by the National Cancer Institute (NCI) to answer the need for a standardized format for data collection and storage of clinical trials regarding cancer [19]. Early implementation of CDEs can be observed within the Cancer Informatics Infrastructure [20]. A set of software known as caCORE has been developed to bring together data from various sources to a centralized database. Within caCORE resides the Cancer Data Standards Repository (caDSR), a metadata registry for CDEs. The caDSR is a database supported by the National Cancer Informatics Program that stores these CDEs [21]. Implementation of the caDSR utilizes the ISO/IEC 11179 standard for metadata registries [22]. The ISO/IEC 11179 describes a model for formally associating data model elements with their intended meaning. In the ISO/IEC 11179, a data element is defined as a unit of data for which the definition, identification, representation, and permissible values are specified by means of a set of attributes [22]. The ISO 11179 standard allows the system to determine that two data elements from two different models are alternative representations of the same real world entity [23,24]. The ISO/IEC 11179 specifies an information model by which CDEs are formed and stored within the caDSR by means of a structure based on object and property classes. Although these CDEs provide a useful mechanism to formalize the definitions of intended meaning (ie, data element concepts in the language of ISO/IEC 11179) of a CDE using standard vocabularies (eg, NCI Thesaurus, NCIt), a severe limitation of this representation is the lack of specific semantic relations between the object class annotation and the property class annotation [23,24]. Many times, the object class is simply a plain list, a collection of concept code annotations without semantic relations. This lack of a formal semantic representation presents a problem when attempting to study the temporal relationships associated with a data element concept. As a result, very few studies have focused on the formal representation of temporal relationships associated with a data element concept. Although there exist attempts to represent the temporal relationships within CDEs of caDSR, the lack of standardization still results in ambiguity. For example, ambiguities between the preferred definitions, an abbreviated form of the contents of the CDE, can be seen between CDEs. For CDE 2458736, the preferred definition is PILL_QUANT_DT, whereas for CDE 23 it is OTX_DATE, where DT and DATE both refer to the same meaning. Such ambiguity is highly inconvenient when attempting to study temporal relationships via an ontological approach.

### Objective

The primary objective of this research was to represent time-relevant CDEs [22] within the NCI caDSR [25]. Using the Web Ontology Language (OWL) [26] as a technology to model CDEs allows for the leverage of a plethora of reasoning and inference tools available on the Semantic Web. In this paper, we focus on the coverage of patterns developed from the TEO [14], an ontology-based approach to improve semantic representation, on the temporal aspects of CDEs within caDSR.

## Methods

### Materials

#### Cancer Data Standards Repository Common Data Elements

The structure of CDEs can be understood by analyzing each component of the CDE. For the purposes of our study, the following fields were useful: (1) DataElement number, (2) PublicID, (3) LongName, (4) PreferredName, (5) PreferredDefinition, and (6) DataElementConcept. The DataElement number and PublicID were used as identifiers for the CDEs. The LongName and PreferredDefinition fields contained information used in generating TEO patterns, which will be explained later in the paper. The PreferredName and DataElementConcept contain current representations of the CDEs in caDSR with NCIt codes.

With the TEO framework, we investigated its usage in representing the temporal components of the CDEs. By using the various OWL classes of TEO, we can generate *building blocks* whereby temporal components of a CDE can be organized and classified. The *building blocks* can simply be described as the representational patterns in the Resource Description Framework (RDF) triples that are built using TEO. This ultimately affords the creation of parsable and therefore, machine-readable, temporal elements of the CDEs within caDSR.

#### Semantic Web and Web Ontology Language

**Figure 1:**
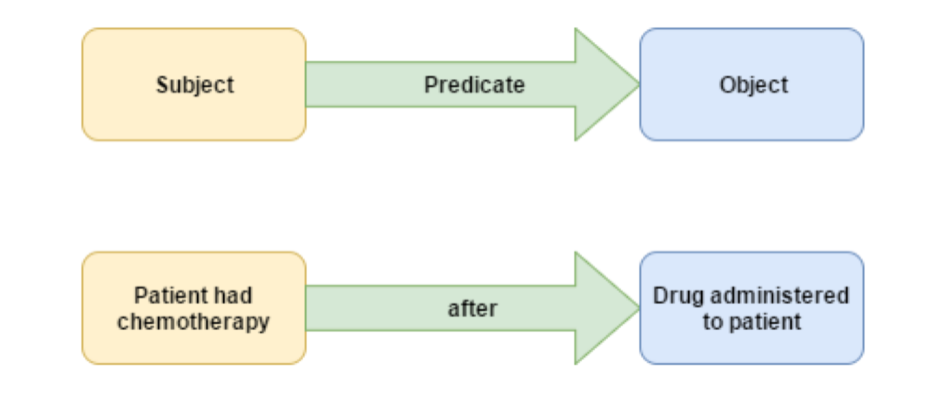
Resource Description Framework (RDF) triple example.

Our efforts focused on addressing the issues regarding (1) giving structure to the vastly unstructured data within the CDEs, (2) capturing temporal relationships between events in the CDEs stored within caDSR, and (3) organizing the data to be machine-readable and processable as opposed to simply being human-readable. To achieve these goals, we took advantage of the Semantic Web and OWL [26]. By representing the temporal dimension with OWL, many of the reasoning capabilities available on the Semantic Web can be leveraged. The temporal relationships themselves can be annotated using an ontology and stored as RDF triples (Figure 1) [27].

#### Time Event Ontology

**Figure 2:**
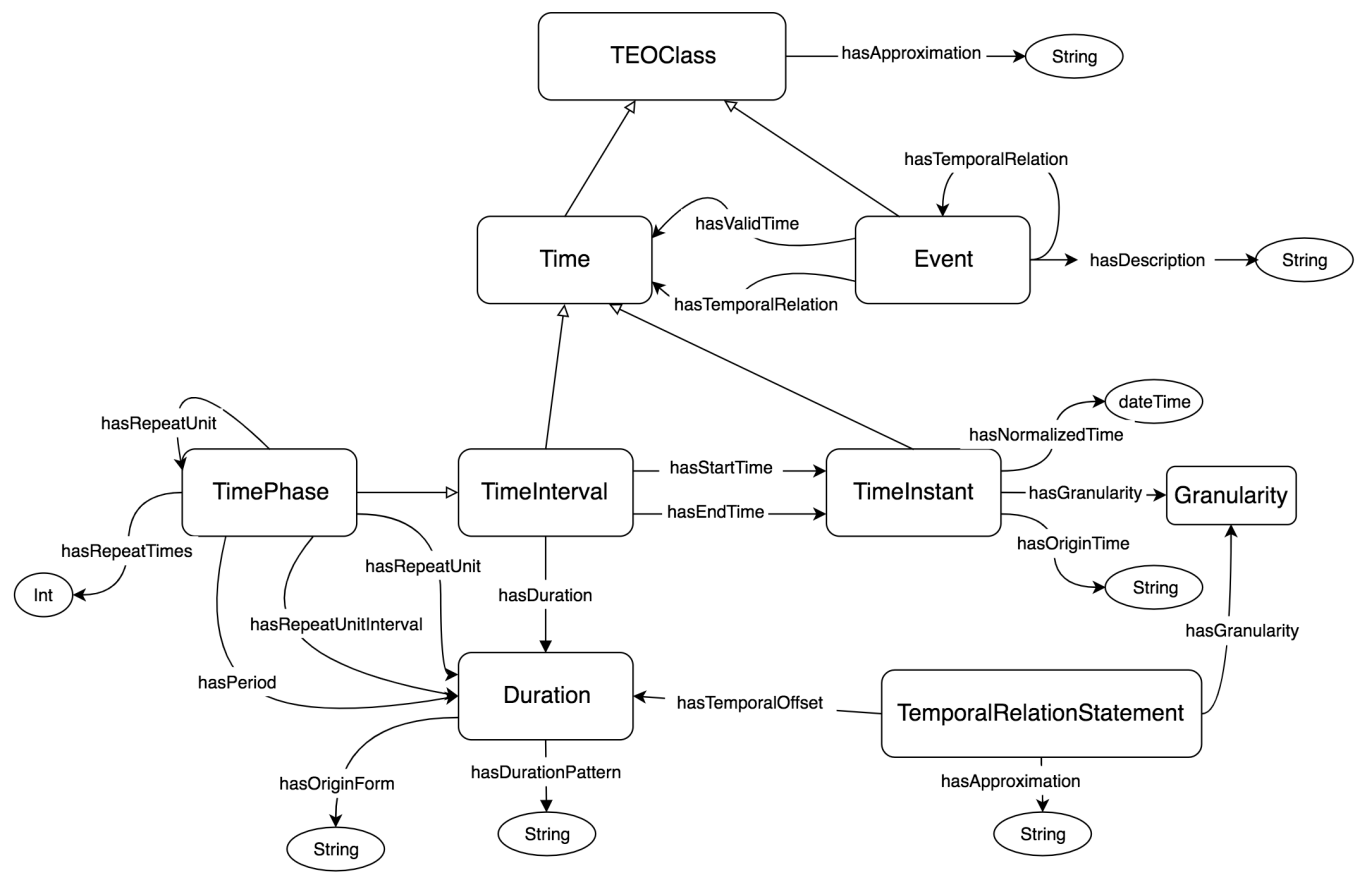
Graphical representation of Time Event Oncology (TEO).

TEO is an ontology designed for a formal conceptualization of time-related information (eg, temporal expressions, temporal relations, and granularities of time) in both structured data and textual narratives. The design of TEO was based primarily on its predecessor, the Clinical Narrative Temporal Relation Ontology (CNTRO), a Semantic Web ontology created for representing temporal relationships within clinical narratives [13]. Although CNTRO was primarily focused on annotating clinical narratives, TEO was designed with the goal of annotating a very general category of temporal relationships. In addition, TEO has been refined to cover more semantic features such as finer level of granularity of temporal relations, standard representations of temporal durations, and more sophisticated representations for reoccurred events. The general architecture of TEO can be seen in Figure 2.

To understand how TEO patterns are generated, an elementary understanding of the components of TEO is required. The following section presents a brief overview of the components and their meanings to lay the groundwork for understanding the TEO patterns used to represent the temporal component of the CDEs.

TEO is composed of the following OWL classes: *Event, Time, TimeInstant, TimeInterval, TimePhase, Duration, Granularity,* and *TemporalRelationStatement*. Object properties and data properties are also defined to represent relations and attributes of the classes. Additionally, the TEO framework allows various OWL classes to be interconnected via OWL classes that act as predicates.

The *Event* class is simply defined as any occurrence. Each instance of an *Event* can be related to another instance of *Event* via the *hasTemporalRelation* property or to an instance of the *Time* class via the *hasValidTime* or *hasTemporalRelation* property. The detailed temporal relations in TEO are defined and extended on top of Allen’s temporal algebra [28].

The *Time* class is defined as a superclass of the *TimeInstant* and *TimeInterval* classes. A *TimeInstant* can be conceptualized by any event that can be represented by a discrete time point within a given time line. For example, “28 APR 2017” can be represented by *TimeInstant.* The granularity of the *TimeInstant* can be represented using the object property *hasGranularity* with domain *Granularity* that defines a predefined set of temporal granularities, including seconds, minutes, days, etc. A *TimeInterval* can be connected to two instances of *TimeInstant* that represent the start time and end time via the *hasStartTime* and *hasEndTime* properties. Additionally, the duration of the *TimeInterval* can be represented with the *Duration* class. For example, in “Around 06 APR 2017, the infant developed constipation, which persisted as of 28 APR 2017,” the time of “constipation” can be represented by a *TimeInterval.* The duration of this *TimeInterval* (22 days) can be represented by a *Duration* class. The *Duration* class is linked with the properties *hasDurationPattern*, which formally defines each duration. For example, we can use “5D10H” to represent “five days and ten hours.” It is important to note that an instance of *TimeInterval* is not required to have all three components previously listed. However, to be formally defined as a TimeInterval for reasoning purposes, it is required that two of the three components be defined. This allows for the third missing component to be inferred via a reasoner.

Within the *Time* class, the *TimePhase* class is defined as an extension of the *TimeInterval* class with additional properties. *TimePhase* is a special case of the *TimeInterval* and is composed of multiple instances that reoccur periodically. For example, in “Judy has swum 2 hours a day for 6 months,” the “2 hours a day for 6 months” is a *TimePhase* (Textbox 1). The *hasRepeatTime* parameter stores an integer that describes how many times the instances reoccur. The *hasRepeatUnitInterval* property connects to an instance of *Duration* representing the time between two recurring instances. The *hasRepeatUnit* can store either an instance of *Duration* or another *TimePhase*, allowing nesting of multiple *TimePhase* instances. In the above example, the *TimePhase* has the *hasRepeatUnit* property that stores a *Duration* of 2 hours. The *hasPeriod* property connects to an instance of *Duration* representing the sum of the duration between two recurring instances and the duration of the instance. In the above example, the *TimePhase* has the *hasPeriod* property with a *Duration* of 1 day and the *hasDuration* property with a *Duration* of 6 hours. Again, all properties are not required to be specified, but a minimum number is necessary to adequately infer the rest via a reasoner.

Finally, the *TemporalRelationStatement* class is used to add constraints to an RDF triple. It has a built-in *hasApproximation* parameter to account for any temporal uncertainty. For example, in “his constipation may have started before the medication,” the RDF triple [constipation][*before has Approximation: True*][“medication”]. Additionally, it can store an instance of *Duration* within the *hasTemporalOffset* parameter to, for example, specify a duration of time after an event occurs.

**Resource Description Framework (RDF) representation of time phase example. Bold font indicates the class, and italic font indicates the property.**

<tPhase1>            rdf:type  **TimePhase**;

*hasDuration * durat1;

*hasRepeatUnit * durat2;

*hasPeriod * durat3;

<durat1>               rdf:type  **Duration**;

*hasDurationPattern* 6M;

<durat2>               rdf:type  **Duration**;

*hasDurationPattern* 2H;

<durat3>               rdf:type  **Duration**;

*hasDurationPattern* 1D;

### Methods

#### Identifying Temporal Components Within Common Data Elements

It was necessary to retrieve the CDEs that contain a temporal component from the general population of CDEs. Thus, the CDE parser was created and utilized to accomplish this goal. The NCI offers a CDE browser that we used to obtain the CDEs utilized for our analysis. The CDEs were downloaded in .xml format from the CDE browser as of August 4, 2015.

Of the 42,956 CDEs within caDSR that were downloaded, 7369 were identified to have at least one temporal component. This was accomplished using the CDE parser to target certain keywords within the LongName and PreferredDefinition fields. Each keyword was assigned to a particular TEO class, which will hereafter be referred to as building blocks (Table 1). These building blocks help to inform the annotator of the contents of the CDE. The building blocks are output alongside the parsed CDEs. The keywords are regular expressions that allow for a wide range of temporal information to be captured by one keyword. For example, dates (eg, December 31, 2015) can be easily represented using regular expressions. However, in the case of caDSR, these keywords were not found in any of the CDEs. In the future, regular expressions can easily be added to the CDE parser as the need arises.

Textbox 2 provides an example of a CDE that has been extracted using the CDE parser. The aforementioned fields are present along with the building blocks, marked in bold, that partially compose the CDE. In this particular example, the CDE parser identified the keywords “interval” and “date.” These were assigned to the TEO classes TimeInterval and TimeInstant/TimeInterval/Date, respectively. The reason that there are multiple TEO classes assigned to a specific keyword is because of the inherent ambiguous nature of the keywords. These keywords can serve as a guide to the annotator but are primarily used to extract time-relevant CDEs. Because the keywords are simply a guide, this allows the annotator flexibility in assigning the TEO classes and creating the patterns that will be described in the next section of the paper.

#### Time Event Ontology Pattern Generation

To generate the representation patterns, the temporal aspects of the caDSR CDEs in the observing set were manually annotated using TEO as an ontological basis. The patterns were created by taking into account the building blocks identified by the CDE parser, as well as the LongName and PreferredDefinition fields. By taking into account the PreferredDefinition in conjunction with the LongName, it can be assured that the CDE is assigned an appropriate pattern.

Table 2 provides an example of a CDE that has been annotated using a TEO pattern. The TEO patterns can be constructed by having the annotator first look at the LongName field to get a general idea of the content of the CDE. The PreferredDefinition field can be used to confirm the content of the CDE. In this case, the pattern [Event*] [TemporalRelation] [Event] can be used to represent the temporal aspect of the CDE. The TemporalRelation block stores the *before* temporal relation that relates the “treatment type” to the “surgical procedure.” The second Event in the pattern stores the “surgical procedure type.” All unasterisked fields are assumed to be static information defined by the CDE. Static information defined by the CDE is assumed to be constant across all instances of the CDE. In Table 2, the surgery and temporal relation of *before* is considered static information because this information is constant for all instances of the CDE. The starred Event stores the treatment that was given before the surgical procedure type. This starred Event is assumed to be variable based on what kind of information is stored within the CDE, which can change among the different instances of CDEs. TEO does not define any subclasses under the Event class with the assumption that each application of the TEO could further define subclasses that are specific to that domain. In this case, we could define the type of *event1* as Treatment, which is a subclass of Event if needed.

**Table 1:** Keywords represented with regular expressions delimited by commas and their corresponding Time Event Ontology (TEO) class.

Keyword regular expressions	TEO^a^ class (building blocks)
Jan(uary)?,Feb(ruary)?,Mar(ch)?,Apr(il)?,May,June,July,Aug(ust)?,Sept(ember)?,Oct(ober)?,N ov(ember)?,Dec(ember)?,today,morning,night,date	TimeInstant
	TimeInterval
	Date
seconds,minutes?,hours?,days?,weeks?,months?,years?	Granularity
	Duration
before,while,prior to, ago,previous(ly)?,post(- )?,subsequent,concurrent(ly)?,meets?,overlaps?,finish(es)?,starts?,during,after,within,until,when	TemporalRelation
	TimeOffset
recurrent,frequent,intermittent,periodic,repeat(ed)?	TimePhase
Interval	TimeInterval

^a^TEO: Time Event Ontology.

**Example of a common data element (CDE) parsed with the CDE parser. Bold font indicates the class.**

**[TimeInterval, TimeInstant/TimeInterval/Date]**

<DataElement num=“36405”>

<PUBLICID>4199738</PUBLICID>

<LONGNAME>QT Interval Medication Administered Last Date</LONGNAME>

<PREFERREDNAME>4199693v1.0:2192181v1.0</PREFERREDNAME>

<PREFERREDDEFINITION>

information related to the date QT interval medication last administered.

</PREFERREDDEFINITION>

<DATAELEMENTCONCEPT>

<PreferredName>4199691v1.0:2233610v1.0</PreferredName>

</DATAELEMENTCONCEPT>

**Table 2:** Common data element (CDE) annotated with a Time Event Ontology (TEO) pattern. Bold font indicates the class, and italic font indicates the property.

Representation type	Content	
CDE^a^	**[TemporalRelation/TimeOffset]** <DataElement num=“44077”> <LONGNAME>Treatment Given Prior To Surgical Procedure Type</LONGNAME> <PREFERREDDEFINITION>Text term to describe the kind of treatment given to an individual prior to surgery.</PREFERREDDEFINITION>	
TEO^b^ pattern	[Event*] [TemporalRelation] [Event]	
Extended TEO pattern	[Event=Treatment*] [TemporalRelation=before] [Event=Surgical Procedure Type]	
RDF^c^ triple representation	<event1>	rdf:type **Event (Treatment)**; rdfs:label *; *before* <event2>; rdf:type Event; rdfs:label “Surgical Procedure Type”;



	<event2>	

^a^CDE: common data element.

^b^TEO: Time Event Ontology.

^c^RDF: Resource Description Framework.

## Results

### Common Data Element Parser Performance

First, it was important for us to analyze the sensitivity and specificity performance of the CDE parser to confirm that the CDEs extracted actually contained a temporal component. True positive denotes the CDEs correctly identified as containing a temporal component. True negative denotes the CDEs correctly excluded from the time-relevant CDEs. False positive denotes the CDEs that do not contain a time component but were retrieved by the CDE parser. False negative denotes the CDEs that have a time component but were not retrieved by the CDEs.

In our analysis of the CDE parser and TEO pattern performance, we performed two iterations of annotation with the first iteration serving as a pilot set to obtain a general idea of CDE parser and TEO pattern performance and to generate a second set of data with a nonarbitrary sample size. To evaluate the CDE parser performance, we used the data from the second, more statistically robust iteration of annotation. Additionally, two sets of data (n=425) were randomly generated from the population of CDEs that were not retrieved by the CDE parser, referred to as complement sets hereafter. These complement sets were used in our analysis to find potential false negatives. In other words, we hoped to identify CDEs with temporal aspects that were not parsed by the CDE parser. Analyses of these complement sets allowed us to identify true negatives and false negatives. As before, two sets of data were used to test for consistency among the complement sets. Three annotators independently examined the complement sets. These two complementary sets of data were used in conjunction with the two test sets (n=425) from earlier. The results are presented in Table 3. Sensitivity values were calculated using the following equation:


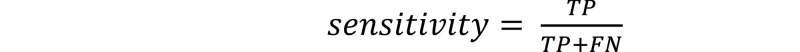


Specificity values were calculated using the following equation:


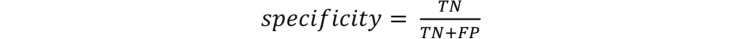


Both sensitivity and specificity parameters exhibit good performance. Interestingly, the sensitivity values are higher on average than the specificity values. This indicates that the performance of the CDE parser could be improved by refining the keywords list to ignore CDEs that do not actually possess a time element.

**Table 3:** Sensitivity and specificity data of common data element (CDE) parser.

Annotator	Test set	True positive	True negative	False positive	False negative	Sensitivity	Specificity
1	1	398	408	27	17	0.959036	0.937931
	2	404	415	21	10	0.975845	0.951835
2	1	394	418	31	7	0.982544	0.930958
	2	394	412	33	13	0.968059	0.925843
3	1	391	414	34	11	0.972637	0.924107
	2	397	418	28	7	0.982673	0.93722

### Time Event Ontology Pattern Evaluation

#### Interannotator Agreement

Because the test sets were annotated independently by three annotators, it was necessary to examine the interannotator agreement between the patterns assigned by the three annotators (Table 4). In analyzing the interannotator agreement, the CDEs could be categorized into one of three categories: (1) no difference, meaning that all three annotators assigned the same or equivalent pattern to the CDE; (2) one difference, meaning that two annotators assigned the same or equivalent pattern to the CDE, but one annotator assigned a different pattern; or (3) all different, meaning that all three annotators assigned a different pattern to the CDE.

The CDEs assigned to the one difference category are simply assigned to the pattern that two out of the three annotators used. Upon examination of the CDEs that fall under this category, it was found that the intended meaning behind many of these CDEs were very similar. For example, two annotators annotated a CDE as a TimeInstant, whereas one annotated a CDE as a TimeInstant as an end time of a TimeInterval. However, because the patterns were not exactly identical, they are considered to fall under the *one difference* category. At the root of this discrepancy is likely the misinterpretation of the CDE because of the lack of expertise regarding the contents of the CDE. A domain expert or the creator of the CDE would easily solve this ambiguity problem.

With three differences there exists the problem of being unable to assign a pattern to the CDE because of all the annotations being different. These CDEs would require a domain expert to properly annotate them. We see from the data that the vast majority of the CDEs can be assigned a pattern either by having no difference in the pattern assigned by the annotators or by having two differences whereby the pattern that is used by the majority of annotators is used.

#### Time Event Ontology Pattern Coverage

We were interested in analyzing the coverage of TEO patterns on a randomly generated set of CDEs with a temporal aspect. From the 7369 CDEs identified to have a temporal aspect, we chose to generate a pilot set with an arbitrary size of n=600. The pilot set was randomly partitioned into an observing set of n=300 and three test sets of n=100. Three sets of n=100 were generated to test for consistency of pattern occurrence among the three test sets. The observing set was used to produce a variety of TEO patterns that could be applied to the CDEs in the test sets. CDEs within the test sets could fall into one of four categories: existing pattern, new pattern, not time-related, and nonrepresentable with TEO (Table 5). The approximate proportion of each classification of CDEs within caDSR is also shown in Table 5 based on our analysis. CDEs that can be represented with a pattern generated from the observing set are *existing patterns*. If a CDE cannot be represented by any of the patterns generated in the observing set, but a new pattern can be generated to represent that CDE, then it is classified as a *new pattern*. On the other hand, the other two sections represent CDEs that are either not time-related at all (*not time-related*), a fault of the parser, or nonrepresentable by TEO because of shortcomings in TEO (*TEO cannot represent*).

The initial pilot set of n=600 was used for two reasons: (1) to train annotators on how to annotate the CDEs with TEO and (2) to garner a general idea of how well TEO can represent the various temporal aspects of the CDEs. The analysis from the first pilot set allowed us to generate a new set of data with a nonarbitrary sample size. We chose to look at the robustness of the CDE parser by analyzing the sensitivity of the parsed CDEs. The sensitivity values were then used to calculate the sample size of the second test set. For the purposes of the calculation, the existing pattern and new pattern sections in Table 5 are important. The existing pattern section is denoted as *true positive* because it is for CDEs that are captured by the manually derived existing patterns. The new pattern section is denoted as *false negative* because the pattern would have been designated as nonrepresentable based on the existing patterns. Thus, true positive and false negative can be used to calculate sensitivity. It should be noted here that we did not calculate specificity in the context of TEO pattern coverage. This is because of the four categories presented in Table 5, none of them fall into the category of a *false positive*. This would result in a trivial specificity value of 1 for all test cases. Thus, we chose to simply utilize sensitivity in the TEO pattern coverage analysis. Additionally, we can utilize the *existing pattern*, *new pattern*, and *TEO cannot represent* sections of Table 5 to calculate the coverage rate of TEO for time-related CDEs using the following equation:


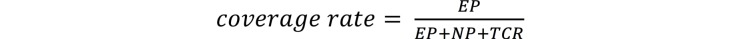


The coverage rate is calculated for the final test sets later in the paper.

Table 6 presents the results of three annotators on the three different test sets within the initial pilot set. The margins of error were calculated using the following equation:

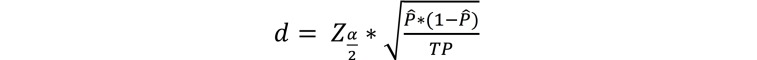



 where *TP* denotes the number of true positive instances.
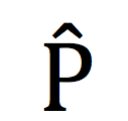
is the sensitivity expressed in decimals. *Z*_α/2_ the z-value, which in our case is 1.96, representing a 95% CI. The sensitivity values, as well as the margins of error between the data sets and annotators are insignificantly different. Thus, we were able to use these results to generate a second test set.

**Table 4:** Interannotator agreement data (N=425).

Test set number	No difference, n (%)	One difference, n (%)	All different, n (%)
1	279 (65.6)	133 (31.2)	13 (3.0)
2	258 (60.7)	146 (34.3)	21 (4.9)

**Table 5:** Test set common data element (CDE) categorization (N=300).

Category		n (%)
**Representable CDEs**^a^		
	Existing pattern	263 (87.7)
	New pattern	9 (2.9)
**Nonrepresentable CDEs**		
	Not time-related	20 (6.8)
	TEO^b^ cannot represent	8 (2.6)

^a^CDE: common data element.

^b^TEO: Time Event Ontology.

**Table 6:** Pilot set annotation results.

Annotator	Test set number	Number of TP^a,b^	Number of FN^c,d^	Sensitivity	Margin of error
1	1	85	2	0.977	0.032
	2	82	5	0.943	0.050
	3	83	9	0.902	0.064
2	1	86	5	0.945	0.048
	2	82	7	0.921	0.058
	3	77	11	0.875	0.074
3	1	89	3	0.967	0.037
	2	84	8	0.913	0.060
	3	83	9	0.900	0.064

^a^TP: true positive.

^b^Denotes the number of true positive instances.

^c^FN: false negative.

^d^Denotes the number of false negative instances.

The following equation was used to obtain a sample size from the sensitivity and margin of error values:

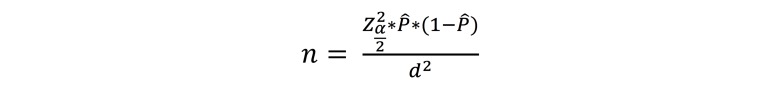



*Z*_α/2_ is, again, the z-value, which in our case is 1.96, representing a 95% CI.
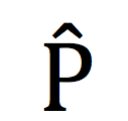
, again, is the sensitivity expressed in decimal form. From the previously mentioned equation, *d*, the margin of error was calculated. On the basis of the data in Table 6, the lowest sensitivity and margin of error were used in the equation for calculating sample size. This results in the largest sample size and subsequently a more representative sample of the population of parsed CDEs. The resultant sample size was n=410 and was rounded to n=425 to yield a more rounded number while still preserving the representative sample size. By rounding up, we are able to preserve the representative sample size, whereas rounding down would result in a less representative sample size. An observing set of n=600, determined by doubling the size of the previous observing set, and two test sets of n=425 were randomly generated from the population of time-related CDEs retrieved by the CDE parser. We note here that it is not necessary to determine the size of the observing set through statistics as it is merely collecting patterns for use in the test sets. The annotation process was repeated to gather TEO coverage data from a population of parsed CDEs with a statistically significant sample size [29].

We present the results of the second iteration of annotation in Table 7. The arithmetic mean of the coverage rates is 94.2% (801/850). In the larger, more representative test set with a statistically significant sample size, the coverage rate of TEO for the time-related CDEs was greater than 90% for all test sets, demonstrating TEO’s effectiveness at representing the time-related CDEs parsed. Additionally, these values have a low spread and are consistent with each other. This demonstrates that a high proportion of the time-relevant CDEs that were retrieved by the CDE parser are representable by TEO patterns.

**Table 7:** Statistically significant test set results.

Annotator	Test set number	Coverage rate
1	1	0.950
	2	0.940
2	1	0.949
	2	0.913
3	1	0.964
	2	0.935

**Table 8:** Most frequently used Time Event Ontology (TEO) patterns used in the observing set of N=600, averaged over three annotators.

Rank	TEO^a^ pattern	n (%)
1	[Event (hasValidTime=[TimeInstant (hasGranularity, hasOrigTime*)])]	186 (31.0)
2	[Event* (hasValidTime=[TimeInterval (hasEndTime=[TimeInstant (hasOrigTime)], hasDuration=[Duration (hasDurationPattern)])])]	117 (19.5)
3	[Event (hasValidTime=[TimeInstant (hasNormalizedTime*)])]	90 (15.0)
4	[Event*] [TemporalRelation] [Event]	42 (7.0)
5	[Event (hasModality*)] [TemporalRelation] [Event]	35 (5.9)
6	[Event (hasValidTime=[TimeInterval (hasEndTime=[Time Instant (hasGranularity,hasOrigTime*)])])]	32 (5.4)
7	[Event (hasValidTime=[TimeInterval (hasStartTime=[Time Instant (hasGranularity,hasOrigTime*)])])]	26 (4.4)
8	[Event* (hasModality*,hasValidTime=[TimeInterval(hasEndTime=[TimeInstant(hasOrigTime)], hasDuration=[Duration(hasValue,hasUnit)])])]	25 (4.2)
9	[Event (hasValidTime=[TimeInterval(hasDuration=[Duration(hasDurationPattern*)])])]	17 (2.8)
10	[Event(hasValidTime=[TimeInterval(hasStartTime=[TimeInstant(hasOrigTime*)],hasEndTi me=[TimeInstant(hasOrigTime*)])])]	11 (1.8)

^a^TEO: Time Event Ontology.

#### Pattern Frequency

While annotating the CDEs with TEO patterns, it became quite evident that many of the CDEs could be characterized by a few patterns. Table 8 lists the top ten most-used patterns in the observing set of n=600, accounting for >97% of all CDEs. In conjunction with Table 8 and Table 9 presents a specific example of each pattern with the corresponding RDF format. The first and third most popular patterns are to be expected because many of the CDEs could simply be classified as storing a date or timestamp. The second most frequent pattern stores the many CDEs that store an answer to a question. This pattern is used to represent CDEs that have questions that ask about some occurrence within a past time frame. We believe that this pattern is a testament to the flexibility of the TEO patterns. The different classes of TEO can be manipulated in a variety of ways to represent a wide variety of temporal aspects within CDEs as shown by the top ten most frequent patterns.

**Table 9:** Specific examples in Resource Description Framework (RDF) format of most frequently used Time Event Ontology (TEO) patterns. Bold font indicates the class, and italic font indicates the property.

Rank	PublicID	CDE^a^ LongName	RDF^b^ representation	
1	4614514	Stage IV disease progression platinum-based chemotherapy date	<event1>	rdf:type **Event**;
				rdfs:label “Stage IV Disease
				Progression Platinum-Based
				Chemotherapy”;
				*hasValidTime* <tInstant1>;
			<tInstant1>	rdf:type **TimeInstant**;
				rdf:label “Date”
				hasGranularity *;
				hasOrigTime *;

2	3191975	Patient reported outcome problem dysuria past week severity score 11 point scale	<event1>	rdf:type **Event**;
				rdfs:label *;
				*hasValidTime* <tInterval1>;
			<tInterval1>	rdf:type **TimeInterval**;
				*hasEndTime* tInstant1;
			<tInstant1>	rdf:type **TimeInstant**;
				*hasOrigTime* date_of_CDE;
				*hasDuration* durat1;
			<durat1>	rdf:type **Duration**;
				*hasDurationPattern* 1 week;

3	3100972	Customer request laboratory final approval date java.util. date	<event1>	rdf:type **Event**;
				rdfs:label “Customer Request
				Laboratory Final Approval”;
				*hasValidTime* <tInstant1>;
			<tInstant1>	rdf:type **TimeInstant**;
				hasNormalizedTime *;

4	2683245	Breast conservation treatment post neoadjuvant therapy not attempt specify	<event1>	rdfs:label *;
				rdf:type **Event**;
				*after* < event2>;
			<event2>	rdf:type **Event**;
				rdfs:label “Neoadjuvant Therapy”;

5	3387810	Maintenance therapy prior recurrent disease discontinue indicator	<event1>	rdf:type **Event**;
				rdfs:label “Maintenance Therapy
				Discontinue”;
				*hasModality* *;
				*before* < event2>;
			<event2>	rdf:type **Event**;
				rdfs:label “Recurrent Disease”;

6	2790	Partial response observed end date	<event1>	rdf:type **Event**;
				rdfs:label “Partial Response
				Observed”;
				*hasValidTime* <tInterval1>;
			<tInterval1>	rdf:type **TimeInterval**;
				*hasEndTime* tInstant1;
			<tInstant1>	rdf:type **TimeInstant**;
				hasGranularity *;
				hasOrigTime *;

7	1157	Prior RT begin date	<event1>	rdf:type **Event**;
				rdfs:label “RT”;
				*hasValidTime* <tInterval1>;
			<tInterval1>	rdf:type **TimeInterval**;
				rdf:label “Prior”;
				*hasStartTime* tInstant1;
			<tInstant1>	rdf:type **TimeInstant**;
				hasGranularity * **;**
				hasOrigTime *;

8	4609733	FACT-Cog Questionnaire version 3 CogPM1 how true past seven days have been able to remember things score 5 point scale	<event1>	rdf:type **Event**;
				rdfs:label *;
				hasModality *;
				*hasValidTime* <tInterval1>;
			<tInterval1>	rdf:type **TimeInterval**;
				*hasEndTime* tInstant1;
			<tInstant1>	df:type **TimeInstant**;
				r *hasOrigTime* date_of_CDE;
				*hasDuration* durat1;
			<durat1>	rdf:type **Duration**;
				*hasDurationPattern* 7 days;

9	3190457	Person clinical study assignment follow-up month duration	<event1>	rdf:type **Event**;
				rdfs:label Personal Clinical Study
				Assignment Follow-up;
				*hasValidTime* <tInterval1>;
			<tInterval1>	rdf:type **TimeInterval**;
				*hasDuration* durat1;
			<durat1>	rdf:type **Duration**;
				*hasDurationPattern* *;

10	3177036	Adverse event outcome assessment observation performed study activity actual date and time range ISO21090.IVL.TS.DATETIME.v1.0	<event1>	rdf:type **Event**;
				rdfs:label “Adverse Event Outcome
				Assessment Observation Performed
				Study Activity”
				*hasValidTime* <tInterval1>;
			<tInterval1>	rdf:type **TimeInterval**;
				*hasStartTime* tInstant1;
				*hasEndTime* tInstant2;
			<tInstant1>	rdf:type **TimeInstant**;
				*hasOrigTime* *;
			<tInstant2>	rdf:type **TimeInstant**;
				*hasOrigTime* *;

^a^CDE: common data element.

^b^RDF: Resource Description Framework.

## Discussion

### Comparison With Current Standard Representation in Cancer Data Standards Repository

It is important to note here that TEO is not intended to replace the current standard representation of CDEs within caDSR but rather enhance the representation of temporal components. Although there is a standard representation of the CDEs already implemented, which is stored in the PreferredDefinition field, it does not consistently represent the temporal components of the CDEs [22]. Table 10 demonstrates some inconsistencies within the standard representation that TEO hopes to address. The temporal components within the Preferred Definition and TEO pattern are bolded. It can be seen that given a CDE with the same TEO pattern, the Preferred Definition field uses a different code to represent the CDE. These inconsistencies are resolved by using the TEO patterns. Thus, it can be seen that the TEO patterns are superior at representing the temporal component of the CDEs.

Within caDSR, there also exist inconsistencies between the LongName field and the PreferredDefinition field in some CDEs. Textbox 3 presents an example of a CDE that has a PreferredDefinition field that is inconsistent with the LongName. The LongName implies that the CDE is an indicator for whether a dental procedure known as a post core is used. However, “post” in the LongName is represented as a temporal relation within the PreferredDefinition field. The TEO patterns would allow for clearer representation of these CDEs by allowing the author of the CDE to designate the post core as an Event to clarify any ambiguities.

**Table 10:** Example standard representation of common data elements (CDEs) versus Time Event Ontology (TEO) patterns.

LongName	PreferredDefinition	TEO^a^ pattern
Off treatment date	OTX_DATE	[Event (hasValidTime=[TimeInstant (hasGranularity, hasOrigTime*)])]
Pills quantity date	PILL_QUANT_DT	[Event (hasValidTime=[TimeInstant (hasGranularity, hasOrigTime*)])]
Therapy prior carmustine administered end date	BNCU_ENDDT	[Event (hasValidTime=[TimeInterval (hasEndTime=[TimeInstant (hasGranularity,hasOrigTime*)])])]
Laboratory data inclusion stop date	LAB_INCL_STOP_DT	[Event (hasValidTime=[TimeInterval (hasEndTime=[TimeInstant (hasGranularity,hasOrigTime*)])])]
Breast conservation treatment post neoadjuvant therapy failed performed reason	BCT_P_NEO_FA_PER_RSN	[Event*] [TemporalRelation] [Event]
Lymph node post neoadjuvant therapy response code	LN_NEOADJ_RESP_CD	[Event*] [TemporalRelation] [Event]

^a^TEO: Time Event Ontology.

**Inconsistencies between LongName and PreferredDefinition field.**

<DataElement num=“28726”>

<PUBLICID>3250740</PUBLICID>

<LONGNAME>Prior Dental Restoration Post Core Use Yes or No Response</LONGNAME>

<PREFERREDDEFINITION>Earlier in time or order._Replacement or reconstruction of a lost tooth structure._Post;

occuring after._The center of an object; indispensable_Use; put into service; make work or employ (something)

for a particular purpose or for its inherent or natural purpose._A caDSR representation term that is used to

indicate a question with permissible values of yes/no</PREFERREDDEFINITION>

### Time Event Ontology Limitations

During the annotation process, we discovered limitations with TEO that prevented complete representation of the temporal aspects within the CDEs. To be specific, events listed as an ordinal number of a series are poorly ontologically represented with TEO. This is because of the fact that TEO requires events to be related to each other via a TemporalRelationship, and simply designating the ordinality of the event is not representable with TEO patterns. Due to this requirement of a relationship between events, other temporal relationships found in the CDEs such as “most recent” or “prior” cannot directly be represented with TEO patterns. However, although these temporal aspects cannot be represented ontologically via TEO patterns, they can still be preserved in the RDF label, allowing a human reader or annotator to see these temporal aspects.

Additionally, irregular series of events are not well-represented by TEO. The TimePhase class was built to handle events that reoccur at a regular interval. Although many events within caDSR and a clinical setting in general reoccur at regular intervals, there are a few CDEs that involve events that reoccur irregularly. For example, given caDSR stores many CDEs that are related to cancer, the recurrence of cancers in a patient can be quite sporadic, making it difficult for us to represent the CDE with TEO. Although the current version of TEO cannot adequately represent these temporal aspects, it is anticipated that a future version of TEO will be able to address these shortcomings.

### Conclusion and Future Direction

As stated before, we are facing an increasing volume of data within the EHR. To keep up with this exponential growth of data, a machine-readable annotation is necessary. The underlying OWL-based representation of TEO allows for the leveraging of many reasoning tools on the Semantic Web. With respect to the CDEs within caDSR, steps have already been taken toward standard representation of CDEs. However, aforementioned shortcomings of the current representation open the door for improvements. TEO provides a valid solution to improving the representation of the temporal components of the CDEs. Although it is not perfect quite yet, given its inability to represent certain temporal aspects, it improves upon the current standard representation of the temporal dimension. We hope to improve upon TEO to allow it to more completely represent the temporal dimension. Additionally, querying of these TEO patterns using SPARQL Protocol and RDF Query Language , Semantic Web Rule Language, and a TEO querier for TEO is a future goal. Ultimately, we hope to develop methods to automatically match the CDEs with patterns. Improvements on the CDE parser to improve the sensitivity and specificity will aid in assigning a TEO pattern to the time-relevant CDEs within caDSR. This can be done by refining the keywords list that is used to retrieve the CDEs, as well as incorporating standardized concept codes. In conjunction with these improvements, actual implementation into caDSR to represent the time components of CDEs is our ultimate goal. By improving upon the representation of the temporal components of these CDEs, we believe that research involving the temporal aspect of CDEs in caDSR will become more efficient.

## Abbreviations

caDSR: Cancer Data Standards Repository
CDE: common data element
CNTRO: Clinical Narrative Temporal Relation Ontology
EHR: electronic health record
FN: false negative
NCI: National Cancer Institute
OWL: Web Ontology Language
RDF: Resource Description Framework
TEO: Time Event Ontology
TP: true positive
